# Sleep Quality, Sleep Hygiene Behaviors, and Psychosocial Determinants Among Undergraduate Students in South‐Eastern Bangladesh: A Cross‐Sectional Study

**DOI:** 10.1002/puh2.70262

**Published:** 2026-05-05

**Authors:** Md. Mayin Uddin Hasan, Intesar Mahmud Sayeed, Mohammed Zahidul Islam, Md. Shahin Rana, Md. Aminul Anowar, Shantu Ghosh, Abdullah Al Noman, Mohammed Abu Sayeed, Mohammad Injamul Hoq

**Affiliations:** ^1^ Department of Pharmacy International Islamic University Chittagong, Kumira Chittagong Bangladesh; ^2^ Drug Insides and Disease Epidemiology‐DIDE, Khulshi Chittagong Bangladesh; ^3^ Department of Public Health University of Creative Technology Chittagong Bangladesh

**Keywords:** Bangladesh, psychosocial determinants, sleep hygiene, sleep quality, undergraduate students

## Abstract

**Background:**

Sleep disturbances among university students have increasingly been linked to academic stress, lifestyle choices, and psychosocial factors, all of which elevate the risk of insomnia through the demands of academic work and a propensity for irregular sleep schedules. This study evaluated the determinants of sleep quality and sleep hygiene behaviors among university students.

**Methods:**

A descriptive cross‐sectional study was conducted across five universities in Chittagong, Bangladesh, involving undergraduate students selected via stratified random sampling. Student groups were compared using descriptive statistics, *t*‐tests, Mann–Whitney *U*‐tests, analysis of variance (ANOVA), Kruskal–Wallis tests, and Dunn post hoc tests. Multiple linear regression was performed to evaluate predictors of sleep quality (Pittsburgh Sleep Quality Index [PSQI] score), with *p *< 0.05 as the significance threshold. Data analysis and visualization were performed using Stata SE 18 and R.

**Results:**

Out of 1000 participants, the mean age was 22.05 ± 1.55 years, and 49.6% were classified as poor sleepers (PSQI > 5). Significant gender differences were found (*p* = <0.003), with females reporting poorer sleep quality. PSQI differed across disciplines (*p* = 0.028), especially science versus humanities (*p* = 0.023). Poor sleepers had significantly higher scores on the Sleep Hygiene Index (SHI), Academic Stressors Module (ASM), and Cultural and Social Practices Module (*p* < 0.001). In multivariable regression, female gender predicted poorer sleep (*β* = 0.741, 95% confidence interval [CI]: 0.254–1.228, *p* = 0.003), humanities discipline (vs. science) was also associated with higher PSQI scores (*β* = 0.621, 95% CI: 0.108–1.133, *p* = 0.018), whereas middle‐income status predicted better sleep (*β* = −0.999, 95% CI: −1.813 to −0.185, *p* = 0.016).

**Conclusion:**

Poor sleep quality is prevalent among university students and is primarily influenced by gender, academic discipline, socioeconomic background, and co‐occurring psychosocial stressors. Targeted interventions are needed to improve educational outcomes through sleep hygiene education and context‐specific strategies.

## Graphical abstract

Poor sleep is highly prevalent among university students and is shaped by gender differences, academic discipline, socioeconomic status, and psychosocial stress. These findings underscore the need for targeted sleep hygiene and stress management interventions to enhance student health, well‐being, and academic performance.

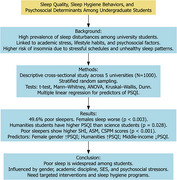

## 1 Introduction

Poor sleep is a prevalent health concern that affects overall well‐being, yet it often goes undiagnosed because it does not meet the criteria for a recognized sleep disorder. It is broadly characterized as inadequate restorative rest, associated with reduced sleep satisfaction, in adults without a clinically defined sleep disorder [1].

Inadequate sleep quality and insomnia are chronic issues affecting the community health of university students. Studies have reported prevalence rates ranging from 9.4% to 38.2%, with a weighted average of 18.5% [2]. Recent research indicates even higher rates, with 65% of students experiencing poor sleep quality and 55% reporting insomnia symptoms [3]. The prevalence of sleep‐related issues in university students has increased significantly over the past decade, rising from 22.6% in 2010 to 30.5% in 2018 [4]. Meta‐analytic studies indicate moderate correlations among sleep quality indicators, insomnia prevalence, and subjective stress among undergraduate students [5]. The prevalence of insomnia, a chronic sleep disorder, has a remarkably high recurrence rate, with about 40% continuing to experience symptoms 5 years later [6]. Research consistently shows a strong relationship between university students’ sleep quality, mental health, and academic performance. A study found that 63.2% of medical students are poor sleepers, and the quality of sleep is highly correlated with depression, anxiety, and stress [7, 8].

Modern patterns of lifestyle behavior significantly affect sleep quality and hygiene. Poor sleep hygiene is one of the risk factors of insomnia, a condition that is linked to poor academic performance [9]. In addition, excessive technology use, particularly before bedtime, disrupts sleep patterns and reduces sleep duration [10, 11]. Social media use and exposure to blue light from electronic screens can affect melatonin release, thereby reducing subjective sleepiness [11]. The youths prioritize technological activities and socialization over sleep, promoting poor sleep patterns and sleep quality. This challenge was exacerbated during the COVID‐19 pandemic, as remote learning increased time spent in front of screens, resulting in decreased academic performance and lower sleep metrics among students [12, 13, 14, 15]. Prolonged screen time before sleep has been repeatedly linked to poor sleep and daytime sleepiness, which in turn affects general productivity [16]. At the same time, several lifestyle factors, including weight gain, lack of physical activity, and caffeine or alcohol use, also play a role in disrupting sleep [10].

Research on sleep patterns in low‐ and middle‐income countries (LMICs) shows substantial heterogeneity in total sleep duration and sleep quality, influenced by cultural, environmental, and societal factors. Despite the similarities between aggregate sleep measures in LMIC populations and those of high‐income environments, methodological inconsistencies still preclude definitive interpretation [17]. Environmental factors, such as household cleanliness, noise levels, and neighborhood infrastructure, significantly affect the quality of sleep among South Asian caregivers [18]. Cross‐cultural studies highlight variations in sleep duration and disturbances among young populations, emphasizing the need for culturally sensitive interventions where the reduction in total sleep time that is observed worldwide can be explained by the environmental factors (noise, light pollution, and radio‐frequency exposure) and by the societal factors (transportation and internet use) [19].

Research on sleep quality and mental health among Bangladeshi university students reveals concerning trends, with poor sleep quality prevalence ranging from 59.4% to 92.3% and showing consistent links to anxiety, depression, fear of COVID‐19, and decreased quality of life [20, 21, 22, 23]. Many students (58.4%–73%) report sleeping less than 6 h per day, and 44.9% rate their sleep quality as only fair [24, 25]. Insomnia is also widespread, with 25.5% of entrance examination candidates and 76.5% of university students reporting moderate‐to‐severe symptoms [26, 27]. These sleep disturbances frequently coexist with mental health disorders, as 83.3% of respondents have reported moderate to severe anxiety and 84.7% mild to severe depression, whereas inadequate sleep is further linked to increased anxiety, depression, and low academic achievement [26, 27, 28]. Contributing factors consistently identified across Bangladeshi studies include female gender, sleeping fewer than 7 h, excessive social media and internet use before bedtime, academic stress, socioeconomic status, residential setting, and lifestyle habits such as physical inactivity and caffeine consumption [21, 22, 23, 29].

University students are among the groups with an elevated risk of poor sleep quality due to the interplay of heavy academic assignments, irregular sleep patterns, excessive technology use, and lifestyle factors. In the Chattogram scenario, an urban metropolitan hub and the central hub of higher education, these issues are further compounded by environmental and sociocultural stressors, such as noise pollution, periodic electricity load shedding, and high population density. Although the increased risk of these conditions is well recognized, limited research has examined sleep quality in this context. The research on university students in Chattogram will thus provide an opportunity to explain both generalized determinants and local factors that lead to poor sleep, and as a result, produce evidence that can inform the creation of context‐specific interventions to improve student health, mental well‐being, and academic performance.

This study aims to assess sleep quality, hygiene practices and to examine the impact of mental health and academic stressors on poor sleep among university students. In addition, it also assesses sociodemographic and cultural antecedents influencing sleep patterns in Chattogram, Bangladesh.

## 2 Methodology

### 2.1 Study Design and Settings

A descriptive cross‐sectional study was conducted from November 2024 to June 2025 among undergraduate students from five universities in southeastern Bangladesh, specifically in the Chattogram area. The universities included International Islamic University Chittagong (IIUC), BGC Trust University Bangladesh (BGCTUB), Chattogram Veterinary and Animal Sciences University (CVASU), Southern University Bangladesh (SUB), and University of Science and Technology Chittagong (USTC).

### 2.2 Sampling Technique

Five universities in Chittagong were purposively selected to ensure institutional diversity: IIUC, BGCTUB, CVASU, SUB, and USTC. Within each university, the student population was first stratified along two dimensions: academic discipline (Science, Business, and Humanities) and academic year (1st–4th year), yielding a total of 12 strata per university. Participants were then selected from each stratum using probability‐roportional‐Pto‐Size(PPS), so that the number recruited from each stratum reflected its relative size within the overall university population. This approach ensured that all major discipline‐year combinations were systematically represented. Recruitment was conducted face‐to‐face by trained research assistants, who visited departments during scheduled class breaks and common areas across multiple visits to each institution to achieve the target sample size and minimize nonresponse.

### 2.3 Inclusion and Exclusion Criteria

Undergraduate students aged 18 or older from the selected universities were included in the study. Individuals under 18, those with medical or mental health conditions, cognitive impairments, or those unwilling to participate were excluded.

### 2.4 Sample Size Calculation

A general formula for prevalence estimation was used to determine the required sample size to assess sleep quality among university students. On the basis of prior literature indicating a 50% prevalence of sleep quality issues, a 95% confidence level (*Z* = 1.96), and a 5% margin of error, the minimum required sample size was 384 [22, 23]. The original estimated required sample size was adjusted to 480 participants to account for a nonresponse rate of approximately 20% and potential data loss due to nonresponses. A larger sample size was chosen to ensure sufficient statistical power and, consequently, generalizability. The raw data were collected from 1176 students. A subsequent data cleaning process excluded cases with incomplete or missing values, and a sample of 1000 participants was considered for performing analysis. This number, which exceeds the minimum requirement, increases the accuracy and consistency of the research findings.

### 2.5 Data Collection Tools

Data were collected using a validated, structured questionnaire comprising closed‐ended items adapted from previously published studies [23, 30, 31, 32, 33, 34].

#### 2.5.1 Variables Used

##### 2.5.1.1 Demographic Characteristics

Data collection included information from various demographic variables. The study calculated age by averaging participants at 22.05 years (SD = ±1.55). At most institutions, the areas of study are classified into three broad groupings: Science, Business, and Humanities. The departments within the Science are Computer Science and Engineering (CSE), Civil Engineering(CE), Doctor of Veterinary Medicine (DVM), Pharmacy, and Food Science and Technology (FST). The Bachelor of Business Administration (BBA) and the Accounting, Economics, and Banking (EB) are included in the Business domain. Economics, English Language and Literature (ELL), Law (LLB), and Quranic Sciences and Islamic Studies (QSIS) are found under the Humanities department. Participants were classified by academic year (1st–4th), gender (male and female), marital status (unmarried and married), religion (Muslim, Hindu, and Buddhist), monthly family income (10,000–20,000 BDT, 20,001–40,000 BDT, 40,001–60,000 BDT, >60,000 BDT), residence type (family home, university hall, and others), area of living (urban, rural, and others), and number of roommates (none, 1–3, >3).

##### 2.5.1.2 Pittsburgh Sleep Quality Index (PSQI)

The PSQI, developed by Buysse et al. [30], is a multidimensional tool with robust empirical support for assessing sleep quality. It has been translated into various languages, including English and Bangla [23, 30]. The Sleep Questionnaire measures nine aspects of sleep behavior, the impact of nightly disturbances on daily functioning, and the use of hypnotic agents over a 4‐week period. The tool includes 19 self‐report scales, along with an additional five ancillary questions that must be completed by a cohabiting partner or roommate. The range of PSQI scores is between 0 and 21; scores that exceed 5 correspond to poor sleeping quality, and scores of 5 and below correspond to good sleeping quality [31]. For this study, we provided participants with the previously validated Bangla version of the PSQI to support comprehension. Prior studies validating the Bangla PSQI have reported satisfactory internal consistency (Cronbach's *α* = 0.816). We used the published validated version by Mondal et al., without modification [35].

##### 2.5.1.3 Sleep Hygiene Index (SHI)

The SHI, developed by Mastin et al., is a well‐established tool for assessing behaviors and environmental factors that undermine an individual's sleep hygiene or sleep health [32]. The scale comprises 13 self‐report items, whose scores were evaluated on a 5‐point Likert scale, ranging from 1 (never) to 5 (always), in response to the questions. The scores can range from 0 to 52, and the higher the score, the poorer the quality of sleep hygiene [31, 32]. Although the SHI was administered in English, we provided participants with a Bangla translation for comprehension, developed by the research team through rigorous linguistic validation and cultural adaptation. No published psychometric validation of a Bangla version was identified in the literature; however, in this study, the Bangla SHI demonstrated acceptable internal consistency (Cronbach's *α* = 0.785) in our sample.

##### 2.5.1.4 Cultural and Social Practices Module (CSPM)

The CSPM is a tool developed for use in Bangladesh to assess cultural and social behaviors such as family obligations, late‐night social gatherings (adda), tea‐shop visits, and religious activities (Fajr and Taraweeh prayers) that may influence sleep. The English questions/items were collected and adapted from published articles on cultural factors affecting daily routines and sleep in Bangladeshi populations [36, 37]. The items are rated on a 4‐point frequency scale that reflects the previous month: 0 = not in the past month, 1 = less than once a week, 2 = once or twice a week, and 3 = three or more times a week [33, 34]. We used both English and Bangla versions for data collection. No independent published psychometric validation of the CSPM was identified; however, in this study, the CSPM demonstrated good internal consistency (Cronbach's *α* = 0.861) in our sample.

##### 2.5.1.5 Academic Stressors Module (ASM)

The ASM evaluates academic behaviors that can affect sleep quality. Such actions include completing mandatory tasks before bedtime, studying in marathon sessions before exams, and absenteeism or tardiness due to inadequate sleep. In addition, university libraries that are open 24 h a day encourage late‐night study. Items are scored using the same 4‐point frequency scale as the CSPM [38, 39, 40]. We used both English and Bangla versions for data collection [37, 41, 42, 43]. In this study, the ASM demonstrated acceptable internal consistency (Cronbach's *α* = 0.712) based on our sample.

### 2.6 Data Collection Procedure

The questionnaire was administered face‐to‐face using paper‐based forms to participants randomly selected from various departments across the five universities. Trained research assistants distributed the questionnaires during class breaks or in common areas to minimize disruption and ensure accessibility. Each session began with a brief verbal explanation of the study to groups of participants, followed by the distribution of informed consent forms. Prospective respondents were required to read and sign the informed consent form, which clearly outlined the study's purpose, the voluntary nature of participation, assurances of confidentiality, and institutional data‐protection procedures. Only those who provided written consent were permitted to complete the questionnaire. The entire process adhered to ethical guidelines, and no incentives were provided to ensure objectivity and avoid bias. Data collection took place over multiple visits to each university, accommodating responder schedules and achieving the target sample size.

### 2.7 Statistical Analysis

Data analysis was performed using STATA SE version 18 (StataCorp, College Station). Descriptive statistics were used to provide a general overview of the participants’ sociodemographic background. The PSQI global score served as the primary outcome measure. Independent *t*‐tests and one‐way analysis of variance (ANOVA) (parametric tests) were used to compare PSQI scores when data were normally distributed; otherwise, Mann–Whitney *U* and Kruskal–Wallis tests (nonparametric tests) were used. The Shapiro–Wilk test was used to assess normality. Significant Kruskal–Wallis outcomes were further examined using post hoc pairwise comparisons with the Dunn test. The factors associated with sleep quality were analyzed using univariable and multivariable linear regression. Variables reaching statistical significance at *p* < 0.05 in the univariable analysis, or judged to be clinically or theoretically important a priori, were included in the multivariable model. The PSQI global score served as the continuous dependent variable. Multicollinearity was assessed using the variance inflation factor (VIF); all VIF values were below 3, indicating no collinearity. Normality of residuals was verified using the Shapiro–Wilk test, and residual plots were inspected for heteroscedasticity. Missing data were addressed through complete‐case analysis: of 1176 questionnaires collected, 176 were excluded due to incomplete or inconsistent responses, yielding a final analytic sample of 1000 participants. Statistical significance was defined as *p* < 0.05 (two‐tailed). Results are reported as beta coefficients (*β*) with 95% confidence intervals (CIs). All graphics and figures were produced using R version 4.3.1.

### 2.8 Ethics Issues

The Institutional Review Board (IRB) of the Department of Pharmacy, International Islamic University Chittagong, Kumira, Chittagong‐4318, Bangladesh, Ref: IRB/Ph‐89(03)/ASA/24, approved the study. The confidentiality of participants’ data was handled with the utmost care. Informed written consent was obtained from all participants, and they participated voluntarily, with a full understanding of the risks and benefits associated with the study. Responses were anonymized, data were stored securely, and only authorized researchers were allowed access to maintain confidentiality.

## 3 Results

A total of 1000 university students participated (mean age 22.05 ± 1.55 years). The sample was predominantly male (58.1%), unmarried (95.2%), Muslim (84.5%), and enrolled in science programs (51.2%). IIUC contributed the largest proportion of participants (39.3%). Most students resided in urban areas (71.5%), lived in family homes (77.4%), and had no roommates (73.7%). The most common family income bracket was 40,001–60,000 BDT (38.0%). Full sociodemographic characteristics are presented in Table 1.

**TABLE 1:** Sociodemographic characteristics of study participants (*N* = 1000).

Variable	Category	Frequency	Percent
**Age**	Mean ± SD (22.05 ± 1.55)		
**Gender**	Male	581	58.1
	Female	419	41.9
	**Total**	**1000**	**100**
**University**	BGCTUB	166	16.6
	CVASU	74	7.4
	IIUC	393	39.3
	SUB	185	18.5
	USTC	182	18.2
	**Total**	**1000**	**100**
**Academic year**	1st year	307	30.7
	2nd year	294	29.4
	3rd year	253	25.3
	4th year	146	14.6
	**Total**	**1000**	**100**
**Field of study**	Science	512	51.2
	Business	138	13.8
	Humanities	350	35.0
	**Total**	**1000**	**100**
**Marital status**	Unmarried	952	95.2
	Married	48	4.8
	**Total**	**1000**	**100**
**Religion**	Muslim	845	84.5
	Hindu	118	11.8
	Buddhist	37	3.7
	**Total**	**1000**	**100**
**Family income (monthly)**	10,000–20,000 BDT	105	10.5
	20,001–40,000 BDT	308	30.8
	40,001–60,000 BDT	380	38
	More than 60,000 BDT	207	20.7
	**Total**	**1000**	**100**
**Residence**	Family home	774	77.4
	University hall	226	22.6
	**Total**	**1000**	**100**
**Area of living**	Rural	285	28.5
	Urban	715	71.5
	**Total**	**1000**	**100**
**Roommates**	1–3 persons	204	20.4
	More than 3 persons	59	5.9
	None	737	73.7
	**Total**	**1000**	**100**
**PSQI category**	Poor sleep	496	49.6
	Good sleep	504	50.4
	**Total**	**1000**	**100**

Abbreviations: BDT, Bangladeshi taka; BGCTUB, BGC Trust University Bangladesh; CVASU, Chattogram Veterinary and Animal Sciences University; IIUC, International Islamic University Chittagong; PSQI, Pittsburgh Sleep Quality Index; SUB, Southern University Bangladesh; USTC, University of Science and Technology Chittagong.

Table 2 shows that group comparisons were performed to explore whether PSQI scores varied significantly across sociodemographic characteristics. An independent samples *t*‐test revealed no significant difference in sleep quality between single (*M* = 6.09) and married participants (*M* = 6.35), *t*(df) = −0.49, *p* = 0.626. Additionally, residence type did not significantly affect sleep quality, *F*(1, 998) = 0.30, *p* = 0.583. However, a Mann–Whitney *U*‐test indicated a significant difference in PSQI scores between male and female participants, with females (rank sum = 223,306) reporting significantly poorer sleep quality than males (rank sum = 277,194), *z* = −3.029, *p* = 0.003. No significant differences in PSQI scores were observed based on area of living (urban vs. rural), *z* = 1.029, *p* = 0.304.

**TABLE 2:** Comparisons of Pittsburgh Sleep Quality Index (PSQI) scores across sociodemographic and institutional variables.

Variable	Categories	Mean/Rank sum (or MS)	Statistic	*p* value
**Marital status**	Single vs. married	6.09 vs. 6.35	*z* = −0.27 (*t* = −0.49)	0.626^a^
**Gender**	Male vs. female	277,194 vs. 223,306	*z* = −3.029	**0.003** ^b^, ^*^
**Area of living**	Urban vs. rural	362,084 vs. 138,416	*z* = 1.029	0.304^b^
**University category**	BGCTUB vs. CVASU vs. IIUC vs. SUB vs. USTC	MS = 8.13	*F* = 0.60, df = 4	0.666^c^
**Academic year**	1st year vs. 2nd year vs. 3rd year vs. 4th year	MS = 17.57	*F* = 1.29, df = 3	0.276^c^
**Religion**	Muslim vs. Hindu vs. Buddhist	MS = 19.23	*F* = 1.41, df = 2	0.244^c^
**Residence**	Family vs. University hall	6.13 vs. 5.98	*z* = 0.15 (*t* = 0.54)	0.583^a^
**Roommates**	1–3 vs. >three vs. none	MS = 11.93	*F* = 0.88, df = 2	0.417^c^
**Field of study**	Science vs. business vs. humanities	—	*χ* ^2^ = 7.148	**0.028** ^d^, ^*^
*Post hoc (Dunn)*	Science vs. business (1–2)	—	*Z* = 1.035	0.902^e^
	Science vs. humanities (1–3)	—	*Z* = 2.660	**0.023** ^e^, ^*^
	Business vs. humanities (2–3)	—	*Z* = 0.848	1.000^e^
**Family income (monthly)**	10,000–20,000 vs. 20,001–40,000 vs. 40,001–60,000 vs. >60,000 BDT	—	*χ* ^2^ (3) = 4.91	0.178^d^

Abbreviations: BDT, Bangladeshi taka; BGCTUB, BGC Trust University Bangladesh; CVASU, Chattogram Veterinary and Animal Sciences University; IIUC, International Islamic University Chittagong; SUB, Southern University Bangladesh; USTC, University of Science and Technology Chittagong.

^a^ Independent samples *t*‐test.

^b^ Mann–Whitney *U*‐test.

^c^ One‐way ANOVA.

^d^ Kruskal–Wallis test.

^e^ Dunn's post hoc pairwise comparison.

* Bold text indicates statistical significance at *p* < 0.05.

A one‐way ANOVA shows that University affiliation, academic year, religion, and roommate status were not significantly associated with PSQI scores in any comparison all *p* > 0.05. A Kruskal–Wallis test identified a statistically significant difference in PSQI scores across fields of study (*χ* ^2^ = 7.148, *p* = 0.028). Post hoc analysis using Dunn's test indicated that students in the science field had significantly different PSQI scores than humanities students (*Z* = 2.660, *p* = 0.023). However, no significant differences were found between science and business students (*Z* = 1.035, *p* = 0.902) or between business and humanities students (*Z* = 0.848, *p* = 1.00). Finally, differences in PSQI scores by family income category were not statistically significant [*χ* ^2^ (3) = 4.91, *p* = 0.178] (Table 2).

**FIGURE 1:**
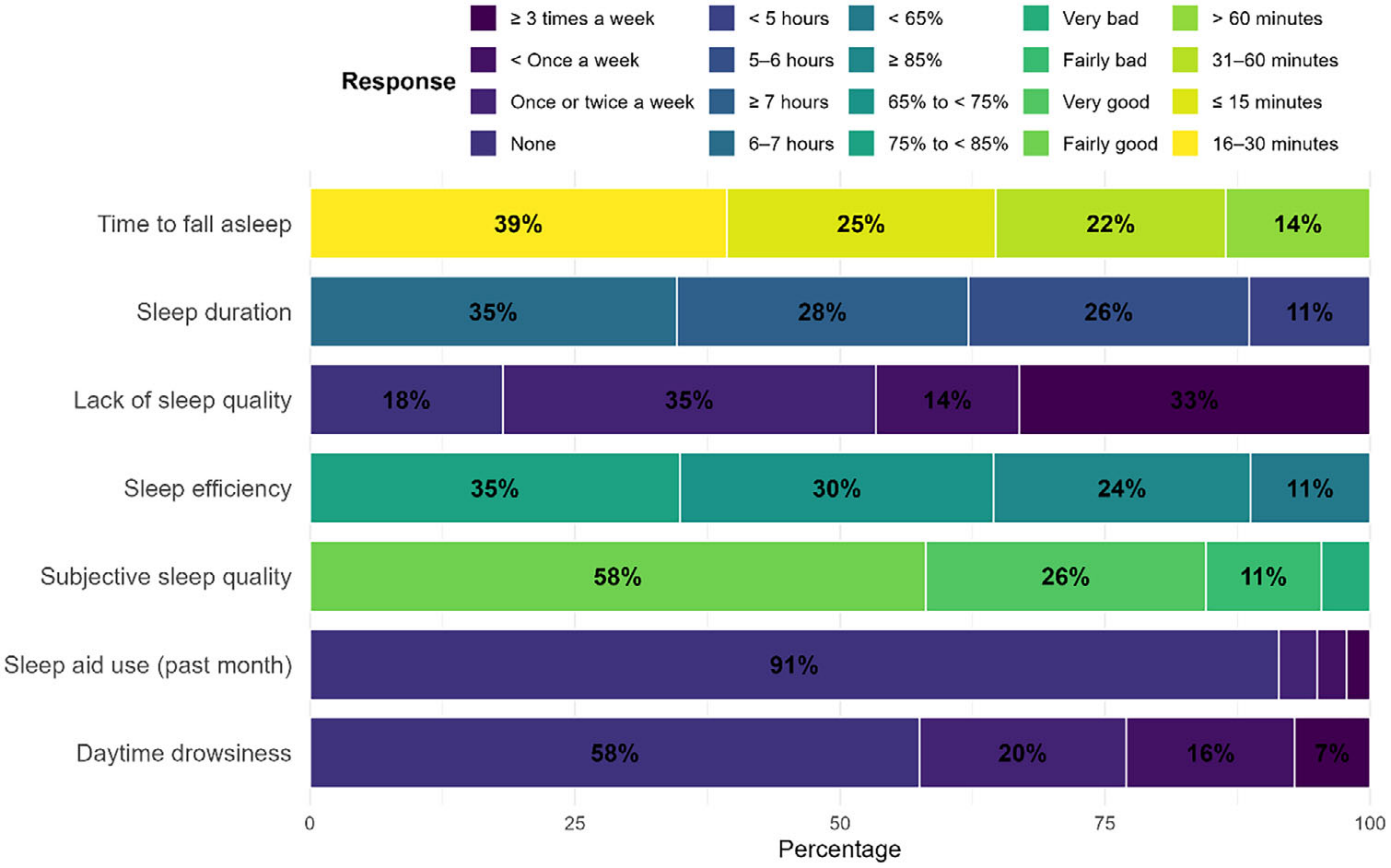
Distribution of PSQI component responses among university students (*N* = 1000).

As shown in Figure 1, the distribution of responses across PSQI components indicates multiple indicators of poor sleep quality among students. The most frequently reported sleep latency was 16–30 min (39.3%), and the majority (62.1%) slept fewer than 7 h per night. One‐third of participants (33.1%) experienced sleep disturbances (i.e., poor sleep quality) at least three times per week, whereas only 18.2% reported no such issues. Sleep efficiency fell below 85% for 75.8% of the sample, and 15.5% rated their sleep as either fairly bad or very bad. Use of sleep aids was uncommon, with 91.4% reporting no use in the past month. However, 42.5% reported daytime dysfunction, such as difficulty staying awake, at least once weekly. These findings underscore the widespread and multifaceted nature of sleep disturbances in this university population (Figure 1).

**FIGURE 2:**
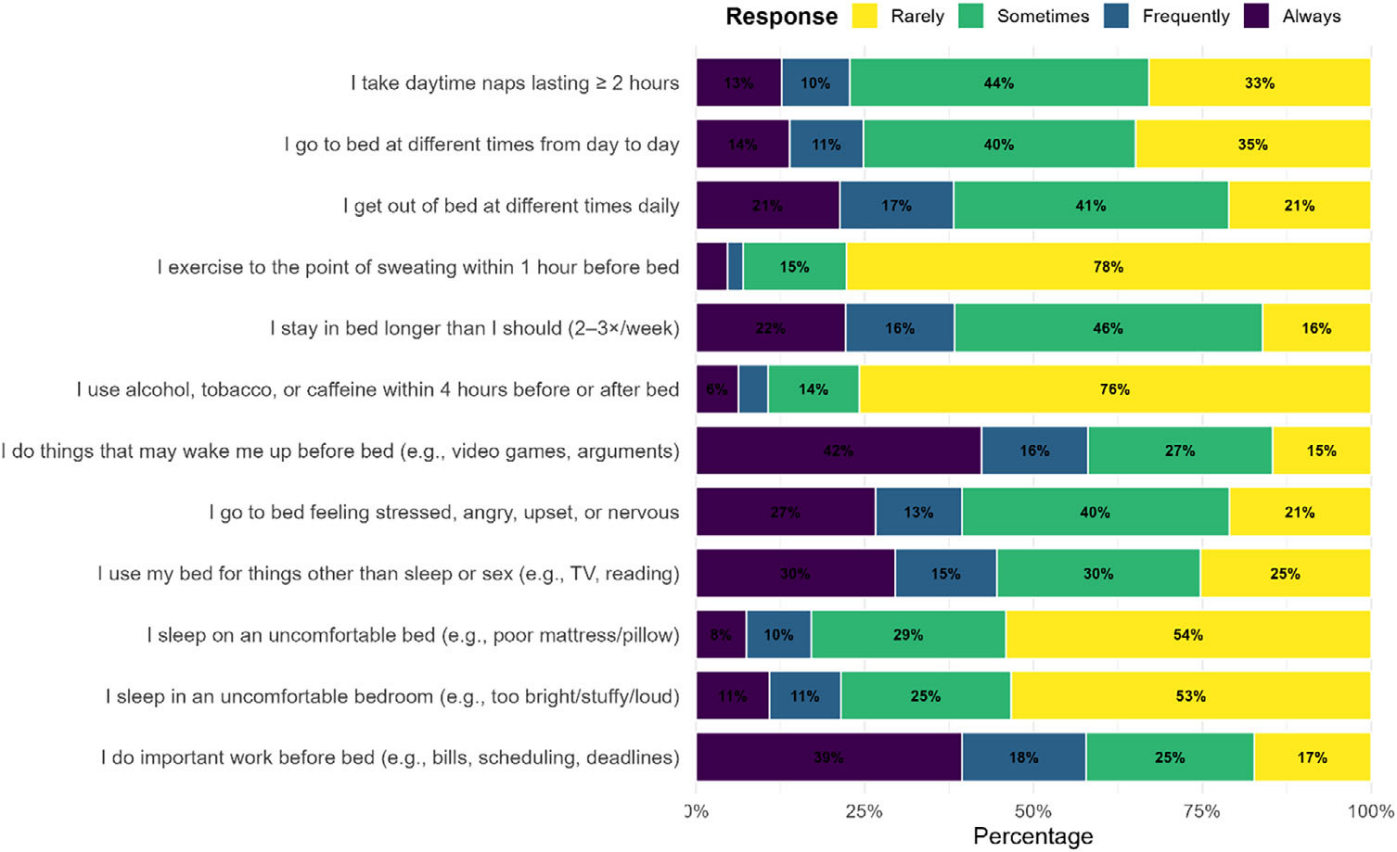
Frequency distribution of responses to Sleep Hygiene Index (SHI) items among university students (*N* = 1000).

Figure 2 displays the response distributions for the 12 SHI items. A notable proportion of students reported poor sleep hygiene behaviors, including inconsistent bedtimes (51.2%) and wake‐up times (38.2%) at least “sometimes.” Over half of participants (57.2%) engaged in arousing pre‐sleep activities (e.g., video games and emotional conversations), whereas 44.5% reported staying in bed longer than necessary multiple times per week. Stress‐related arousal at bedtime was also common, with 58.1% of participants reporting going to bed feeling stressed, angry, or upset. Substance use (alcohol, caffeine, and tobacco) within 4 h of bedtime was infrequent in most respondents (75.8% “rarely”), though 10.7% reported such use “always” or “frequently.” Use of the bed for activities other than sleep or sex was reported by 44.6% at least “frequently.” These findings suggest widespread prevalence of behaviors that may contribute to poor sleep quality in this university population (Figure 2).

**FIGURE 3:**
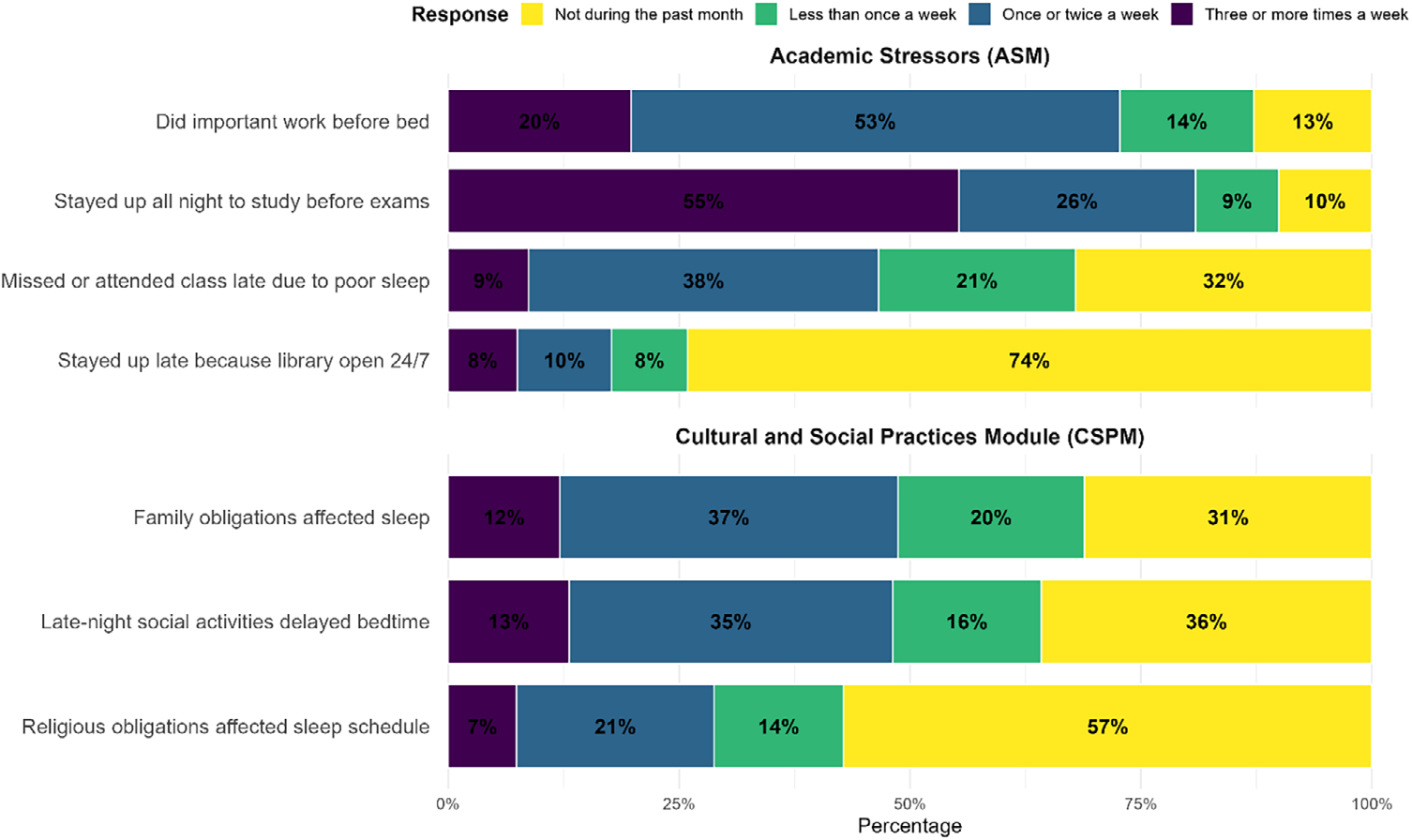
Frequency of sleep disruptions measured by ASM (Academic Stressors Module) and CSPM (Cultural and Social Practices Module) scales.

Figure 3 shows the frequency of factors influencing sleep over the past month, as measured by two Likert‐scale instruments assessing cultural/social and academic influences. Across artistic and social aspects, 311 participants (31.1%) reported no interference from family obligations, 366 (36.6%) experienced interference once or twice a week, and 121 (12.1%) experienced interference three or more times a week. Late‐night social activities delayed bedtime for 350 respondents (35.0%) at least once a week, and religious obligations affected sleep schedules for 214 participants (21.4%) at a similar frequency.

Regarding academic factors, 529 participants (52.9%) reported engaging in meaningful work before bedtime once or twice a week, and 553 (55.3%) indicated staying up all night to study three or more times a week before exams. Additionally, 379 respondents (37.9%) missed or arrived late to classes due to poor sleep once or twice weekly, whereas 741 (74.1%) reported rarely staying up late because the university library's 24/7. These findings suggest that cultural/social commitments, as well as academic demands, commonly contribute to sleep disturbances within the study population (Figure 3).

**FIGURE 4:**
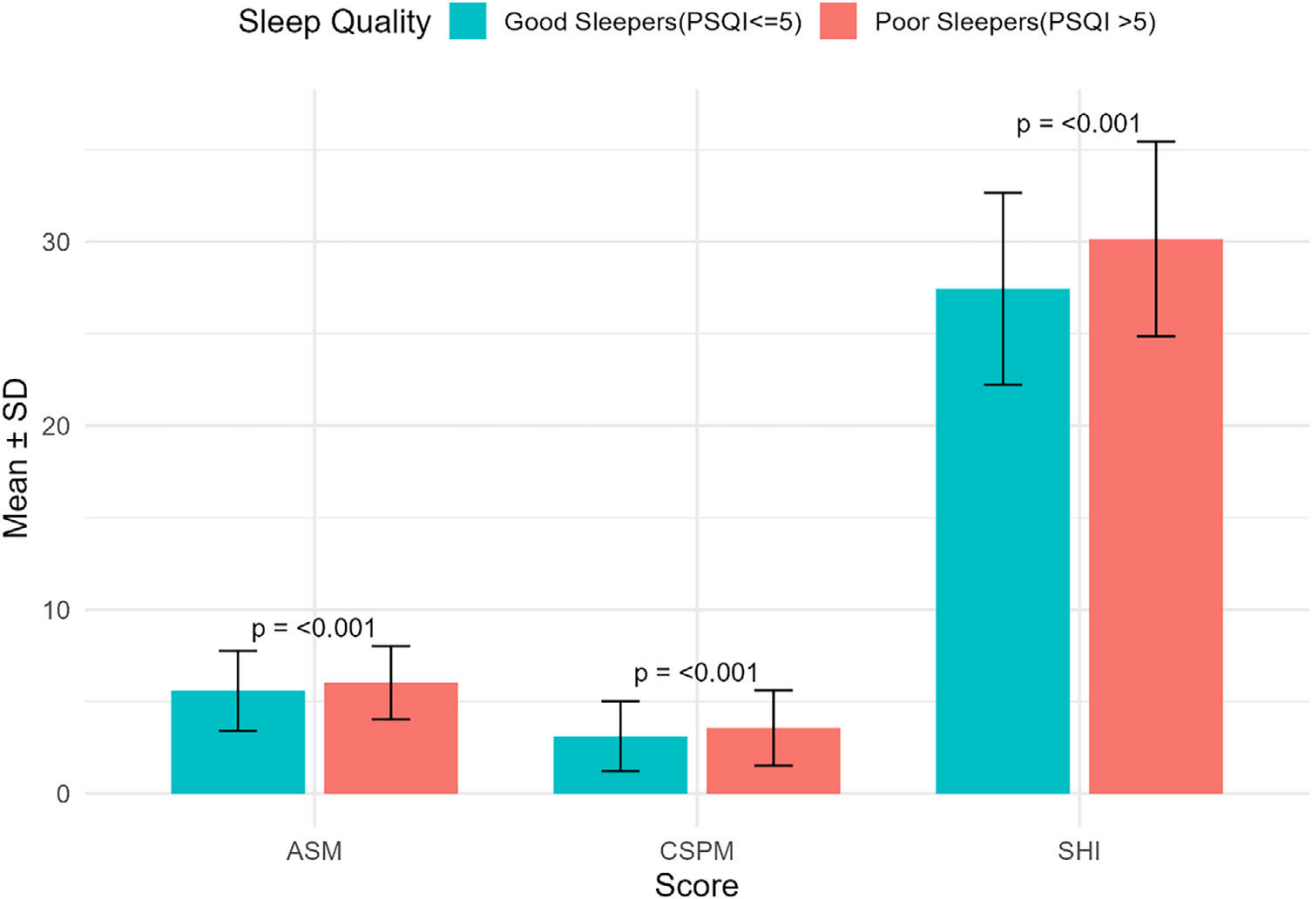
Mean scores of psychosocial and behavioral factors comparing good and poor sleepers classified by the Pittsburgh Sleep Quality Index (PSQI). ASM, Academic Stressors Module; CSPM Cultural and Social Practices Module; SHI, Sleep Hygiene Index.

Figure 4 shows psychosocial and behavioral factors measured by the PSQI. Participants were categorized as good sleepers (*n* = 504) or poor sleepers (*n* = 496) based on their PSQI scores. Mean scores (±standard deviation) are shown for the SHI, CSPM, and ASM. Poor sleepers had significantly higher SHI scores (30.14 ± 5.30) than good sleepers (27.44 ± 5.21), with a mean difference of −2.70 (95% CI: −3.36, −2.05; *t* = −8.14, *p* < 0.001). CSPM scores were also significantly greater among poor sleepers (3.57 ± 2.05) compared to good sleepers (3.12 ± 1.90), difference = −0.46 (95% CI: −0.70, −0.21; *t* = −3.65, *p* = <0.001). Similarly, ASM scores were higher in poor sleepers (6.03 ± 1.99) than good sleepers (5.58 ± 2.18), difference = −0.45 (95% CI: −0.71, −0.19; *t* = −3.42, *p* = <0.001). These results indicate that poor sleepers experienced worse sleep hygiene, greater cultural and social burdens, and higher levels of academic stress (Figure 4).

Univariate and multivariable linear regression analyses were performed to identify factors associated with PSQI scores. In the multivariable model, gender showed a significant association, with female participants reporting higher PSQI scores than males (*β* = 0.741, 95% CI: 0.254–1.228, *p* = 0.003), indicating poorer sleep quality among females. The field of study also showed significant associations. Compared to students in science disciplines, those in humanities had significantly higher PSQI scores (*β* = 0.621, 95% CI: 0.108–1.133, *p* = 0.018), suggesting worse sleep quality. Family income was another significant predictor; participants from families earning between 20,001 and 40,000 BDT (*β* = −0.999, 95% CI: −1.813 to −0.185, *p* = 0.016) and 40,001–60,000 BDT (*β* = −1.023, 95% CI: −1.817 to −0.229, *p* = 0.012) had significantly lower PSQI scores compared to those in the lowest income group (10,000–20,000 BDT), indicating better sleep quality in these middle‐income categories. Age, university affiliation, academic year, marital status, religion, residence, area of living, and number of roommates were not significantly associated with PSQI scores in either univariate or multivariable analyses (Table 3).

**TABLE 3:** Factors associated with Pittsburgh Sleep Quality Index (PSQI) scores among university students.

Variable	Category	*β* (95% CI) (univariate)	*p* value	*β* (95% CI) (multivariable)	*p* value
**Age**		0.076 (−0.071, 0.224)	0.309	0.109 (−0.045, 0.263)	0.167
**Gender**	Male	Reference	—	Reference	—
	Female	0.697 (0.235, 1.159)^*^	**0.003**	0.741 (0.254, 1.228)^*^	**0.003** ^*^
**University**	BGCTUB	Reference	—	—	—
	CVASU	0.349 (−0.301, 0.999)	0.292	—	—
	IIUC	0.204 (−0.442, 0.850)	0.535	—	—
	SUB	0.419 (−0.252, 1.090)	0.221	—	—
	USTC	−0.085 (−1.004, 0.833)	0.856	—	—
**Academic year**	1st year	Reference	—	—	—
	2nd year	−0.128 (−0.718, 0.463)	0.672	—	—
	3rd year	0.450 (−0.164, 1.065)	0.151	—	—
	4th year	−0.077 (−0.805, 0.651)	0.836	—	—
**Field of study**	Science	Reference	—	Reference	—
	Business	−0.406 (−1.098, 0.286)	0.25	−0.397 (−1.091, 0.297)	0.262
	Humanities	−0.773 (−1.274, −0.273)^*^	**0.002**	−0.621 (−1.133, −0.108)^*^	**0.018** ^*^
**Marital status**	Single	Reference	—	—	—
	Married	0.266 (−0.806, 1.338)	0.626	—	—
**Religion**	Muslim	Reference	—	—	—
	Hindu	−0.566 (−1.277, 0.145)	0.119	—	—
	Buddhist	−0.455 (−1.671, 0.761)	0.463	—	—
**Family income (monthly)**	10,000–20,000 BDT	Reference	—	Reference	—
	20,001–40,000 BDT	−0.923 (−1.740, −0.107)^*^	**0.027**	−0.999 (−1.813, −0.185)^*^	**0.016** ^*^
	40,001–60,000 BDT	−0.899 (−1.696, −0.102)^*^	**0.027**	−1.023 (−1.817, −0.229)^*^	**0.012** ^*^
	More than 60,000 BDT	−0.399 (−1.265, 0.467)	0.366	−0.544 (−1.408, 0.319)	0.216
**Residence**	Family home	Reference	—	—	—
	University hall	−0.153 (−0.701, 0.394)	0.583	—	—
**Area of living**	Urban	Reference	—	—	—
	Rural	−0.234 (−0.742, 0.273)	0.365	—	—
**Roommates**	1–3 persons	Reference	—	—	—
	More than 3 persons	−0.431 (−1.502, 0.640)	0.43	—	—
	None	0.185 (−0.388, 0.758)	0.527	—	—

Abbreviations: BDT, Bangladeshi taka; BGCTUB, BGC Trust University Bangladesh; CI, confidence interval; CVASU, Chattogram Veterinary and Animal Sciences University; IIUC, International Islamic University Chittagong; SUB, Southern University Bangladesh; USTC, University of Science and Technology Chittagong.

* Bold text indicates statistical significance at *p* < 0.05.

## 4 Discussion

This study reports a high burden of poor sleep quality among undergraduate students in Bangladesh, with 49.6% reporting poor sleep quality. These findings align with both global and regional evidence on sleep disturbances in student populations. Moreover, sleep quality is associated with several sociodemographic and behavioral factors, such as gender, academic field, socioeconomic status, and cultural traditions.

The 49.6% prevalence of poor sleep quality in this study is slightly lower than the 66.6% prevalence reported among Bangladeshi university students in previous studies [29]. In comparison, it closely aligns with the 52.7% prevalence found in a global meta‐analysis of medical students by Rao et al. [44], highlighting a similar trend in sleep disturbances across different student populations. A study conducted during the COVID‐19 pandemic reported a higher prevalence of poor sleep quality (59.4%), underscoring the significant impact of external stressors, such as the pandemic, on sleep behavior [21]. The comparatively lower prevalence observed in the present study may be attributed to differences in sample composition: The current study focused exclusively on undergraduate students in a specific region, whereas other studies encompassed broader populations or were conducted during periods of heightened stress, such as the COVID‐19 pandemic. Reasons for overall poor sleep quality among students include high academic demands, irregular schedules, excessive screen time, and lifestyle factors such as caffeine consumption and physical inactivity, as evidenced by global studies that show these factors disrupt circadian rhythms and increase sleep latency [45, 46].

Regarding gender, female students showed a significant positive association with PSQI scores compared to male students (*β* = 0.741, *p* = 0.003), consistent with prior research in Bangladeshi and international student samples [29, 47]. This association may be explained by factors including higher levels of reported psychosocial stress and rumination among female students, hormonal variability across the menstrual cycle, and societal role expectations, which align with studies showing females report more sleep disturbances due to rumination and anxiety [48, 49]. However, contrasting evidence from other studies on university students suggests that males exhibit poorer sleep quality (e.g., higher PSQI scores) and greater sleep inconsistency than females, potentially due to factors like later bedtimes from gaming or social media use, or a more intense requirement for regular sleep schedules to maintain quality sleep and academic performance [45, 50].

Students enrolled in humanities disciplines showed significantly higher PSQI scores than those in science disciplines (*β* = 0.621, *p* = 0.018), a finding with limited precedent in the existing literature and a potentially novel contribution [51]. In addition, the significant association between poorer sleep quality and humanities students (*β* = −0.621, *p* = 0.018) aligns with findings in Canadian university students, where, compared to health science students, arts (Humanities) students had worse dysfunctional sleep attitudes, poorer sleep hygiene, and lower sleep quality [52]. However, most prior research had not stratified sleep outcomes by academic discipline [51]. This difference may stem from varying academic structures: Students in the humanities typically face subjective evaluation, heavy essay loads, and more loosely structured schedules, which can lead to procrastination and late‐night studying. In contrast, science students benefit from more regimented lab‐based routines that promote better time management and sleep hygiene [52, 53].

Students from families with middle‐income (earning 20,001–60,000 BDT monthly) reported significantly better sleep than those in lower income brackets (*β *= −0.99, *p* = 0.016; *β* = −1.02, *p* = 0.012), which aligns with the study where reported financial stability was associated with improved sleep quality [54, 55, 56]. The observed association between middle‐income status and better sleep quality may reflect the influence of financial stability on multiple sleep‐related pathways, including reduced financial stress, more conducive living environments, and fewer economic pressures that displace sleep time. These hypotheses are consistent with prior research [57, 58], though longitudinal data would be required to confirm these mechanisms in Bangladeshi student populations.

This study demonstrates significant relationships among ASM, CSPM, and sleep quality, as evaluated using the PSQI. The comparison between groups shows that poor sleepers reported more ASM and CSPM than good sleepers, suggesting that these factors contribute to sleep disturbances. Academic stress findings align with previous studies that have associated academic stress with poor sleep and academic performance [59, 60]. The association between academic stress and poorer sleep quality is consistent with prior studies linking examination pressure and perfectionism to delayed bedtimes and reduced sleep efficiency; however, the present study's cross‐sectional design precludes causal conclusions [60, 61]. Longitudinal evidence suggests that chronic stress may be associated with hypothalamic–pituitary–adrenal axis activation and elevated cortisol levels, which, in turn, are associated with disrupted sleep architecture. However, the directionality of this relationship in student populations remains unclear [60, 61]. According to Mamun et al. [62] and Merikanto et al. [63], cultural and social practices are significantly associated with sleep quality among university students [3, 21].

The present study has several limitations. First, its cross‐sectional design precludes establishing causal relationships. Longitudinal designs would provide a more robust understanding of the temporal relationships between sleep quality and its determinants. Moreover, self‐report measures, such as the PSQI, can introduce recall bias. Objective measures, such as actigraphy, may enhance the validity of sleep measurements.

Second, although the present study employed a multi‐institutional design, its geographic focus on Chittagong may limit the generalizability of findings to other regions or populations. The analysis should be extended to a more diverse geographical area to increase the validity and expand the applicability of the results. Furthermore, using a culturally specific measurement module to assess social and behavioral patterns raises concerns about comparability with data sets collected in other cultural milieus. In this regard, future research should develop assessment tools adaptable to diverse cultural settings.

## 5 Conclusion

In conclusion, poor sleep quality is highly prevalent among university students in Chittagong, Bangladesh, and is significantly associated with gender, academic discipline, socioeconomic status, and cultural practices. These findings underscore the need for culturally appropriate and context‐specific public health interventions, including sleep hygiene education and stress management programs. Such programs have the potential to improve sleep health and, consequently, academic performance. Future studies should use longitudinal designs and include objective sleep measures, such as actigraphy, to better clarify causal mechanisms and support the development of evidence‐based, sustainable interventions.

## Author Contributions

**Md. Mayin Uddin Hasan** conceived and designed the experiments, performed data analysis and interpretation, and wrote the original manuscript draft. **Intesar Mahmud Sayeed** data curation and wrote the original manuscript draft. **Mohammed Zahidul Islam**, **Shantu Ghosh**, **Md. Aminul Anowar**, **Md. Shahin Rana**, and **Abdullah Al Noman** were responsible for data collection and logistical support and contributed to the writing of the manuscript. **Mohammed Abu Sayeed** supervised the study and contributed to the review and editing of the manuscript. **Mohammad Injamul Hoq** supervised the study and contributed to reviewing and editing the manuscript.

## Funding

The authors have nothing to report.

## Ethics Statement

Ethical approval was taken from the IRB of the Department of Pharmacy, International Islamic University Chittagong, Kumira, Chittagong‐4318, Bangladesh, for this work.

## Conflicts of Interest

The authors declare no conflicts of interest.

## Transparency Statement

The corresponding author, Md. Mayin Uddin Hasan, assures that all authors have read and approved the final version of the manuscript; he has full access to all the data in this study and takes complete responsibility for the integrity and analysis of the data.

## Data Availability

The data that support the findings of this study are available from the corresponding author upon reasonable request.
